# Estimation of the Frequency of Intravenous Drug Users in Hamadan City, Iran, Using the Capture-recapture Method

**DOI:** 10.4178/epih/e2012006

**Published:** 2012-10-31

**Authors:** Salman Khazaei, Jalal Poorolajal, Hossein Mahjub, Nader Esmailnasab, Mohammad Mirzaei

**Affiliations:** 1 Department of Epidemiology & Biostatistics, School of Public Health, Hamadan University of Medical Sciences, Hamadan, Iran.; 2 Research Center for Health Sciences, Department of Epidemiology & Biostatistics, School of Public Health, Hamadan University of Medical Sciences, Hamadan, Iran.; 3 Department of Community Medicine, School of Medicine, Kurdistan University of Medical Sciences, Kurdistan, Iran.; 4 Center for Disease Control & Prevention, Vice-chancellor of Health Services, Hamadan University of Medical Sciences, Hamadan, Iran.

**Keywords:** Capture-recapture, Log-linear models, Poisson regression, Intravenous drug users, Iran

## Abstract

**OBJECTIVES:**

The number of illicit drug users is prone to underestimation. This study aimed to use the capture-recapture method as a statistical procedure for measuring the prevalence of intravenous drug users (IDUs) by estimating the number of unknown IDUs not registered by any of the registry centers.

**METHODS:**

This study was conducted in Hamadan City, the west of Iran, in 2012. Three incomplete data sources of IDUs, with partial overlapping data, were assessed including: (a) Volunteer Counseling and Testing Centers (VCTCs); (b) Drop in Centers (DICs); and (c) Outreach Teams (ORTs). A log-linear model was applied for the analysis of three-sample capture-recapture results. Two information criteria were used for model selection including Akaike's Information Criterion and the Bayesian Information Criterion.

**RESULTS:**

Out of 1,478 IDUs registered by three centers, 48% were identified by VCTCs, 32% by DICs, and 20% by ORTs. After exclusion of duplicates, 1,369 IDUs remained. According to our findings, there were 9,964 (95% CI, 6,088 to 17,636) IDUs not identified by any of the centers. Hence, the real number of IDUs is expected to be 11,333. Based on these findings, the overall completeness of the three data sources was around 12% (95% CI, 7% to 18%).

**CONCLUSION:**

There was a considerable number of IDUs not identified by any of the centers. Although the capture-recapture method is a useful and practical approach for estimating unknown populations, due to the assumptions and limitations of the method, the results must be interpreted with caution.

## INTRODUCTION

Illicit drug use is a major public health problem worldwide [1]. It is estimated that about 5% of the world's adult population, or 230 million people, have used an illicit drug at least once in 2010. The number of drug users worldwide is estimated to be about 27 million, which is 0.6% of the world's adult population [2]. About 15.9 million people worldwide may inject drugs, of whom about three million may be human immunodeficiency virus (HIV) positive [3]. Injecting drug use is the main route of transmission for about 10% of HIV infections in the world and 30% of infections outside of sub Saharan Africa. Preventing HIV transmission through injecting drug use is one of the key challenges in reducing the burden of HIV worldwide [1].

It is estimated that between 1.2 and 2 million people are drug abusers in Iran of about 20% to 25% have injected at least once in their lifetime. Approximately 1% of the Iranian population use heroin, of which 40% inject it. Therefore, it is estimated that there are around 280 thousand injecting drug abusers in the country [4].

Hamadan City, the center of Hamadan Province, stands on the north west of Iran and has a population of approximately 600 thousand people [5]. According to the data recorded in the District Health Network database, 1,369 intravenous drug users (IDUs) were identified in the city in 2011, which seemed much less than that expected according to the national estimates.

Illicit drug use is an illegal and stigmatized behavior and most drug users are reluctant to be identified as such. Hence, the number of illicit drug users is prone to underestimation [3]. One method which is commonly used to estimate hard-to-reach populations is the so-called capture-recapture approach. This method was first used in ecology to estimate the unknown size of wild animal populations [6]. It was subsequently used as a potentially useful method in epidemiology for estimating the completeness of ascertainment of disease registers [7,8] and incomplete reporting [9,10]. Actually, the capture-recapture method can principally be applied to any situation where there are two or more incomplete lists.

A high prevalence of HIV [4] and hepatitis C virus (HCV) [11] infections among IDUs emphasizes the importance of quantifying the dynamics of this population. Estimating the size of IDU populations is fundamental for predicting the future burden of HIV and HCV diseases and for planning and providing health care services for this group of individuals [12]. Furthermore, statistically valid and reasonable estimates of IDU prevalence can help policymakers to estimate probable outbreaks of infections associated with drug injection [13]. Populations with negative traits are usually underestimated. Several statistical methods have been suggested for the estimation of such unknown populations [14-16]. A straightforward and easy to use method is the capture-recapture approach. In this study, we used this method as a statistical procedure and a practical means for measuring IDU prevalence by estimating the number of unknown IDUs not registered by any of the three incomplete registries.

## MATERIALS AND METHODS

This study was approved by the Human Subject Review Board of Hamadan University of Medical Sciences and conducted in Hamadan City, the center of Hamadan Province in the west of Iran, in 2012. We extracted the data on IDUs registered in at least one the three centers that provide health services to IDUs in this city, from April 2011 to March 2012. IDUs were defined as people who inject narcotic substances into the body with a hollow needle and a syringe which is pierced through the skin into the body usually intravenously, but also intramuscularly or subcutaneously [17].

There are three centers for providing health care services to IDUs in Iran: (a) Volunteer Counseling and Testing Centers (VCTCs); (b) Drop in Centers (DICs); and (c) Outreach Teams (ORTs). VCTCs provide consultant and educational services to IDUs in order to improve their knowledge of high-risk behaviors and harm reduction methods. In addition, these centers provide diagnostic tests for IDUs and refer the suspected IDUs to specialized medical centers for diagnostic and treatment services. DICs provide social support such as serving food, clothes, and bathing for IDUs. These centers also give agonist and maintenance medications. They also plan educational programs for IDUs in order to improve their knowledge of high-risk behaviors and healthy sexual behaviors. ORTs include a well-trained team of health workers who actively look for the IDUs not referred to VCTCs or DICs. They distribute safety boxes among IDUs including sterile syringes, disinfecting material and condoms. They also encourage IDUs to adopt safe and low-risk behaviors. The IDUs may be identified and registered by any of these centers. Some IDUs may be identified by more than one center. Nonetheless, none of these centers has a complete list of IDUs. We used the capture-recapture method in order to statistically estimate the approximate number of IDUs not identified by these registries.

**Figure 1:**
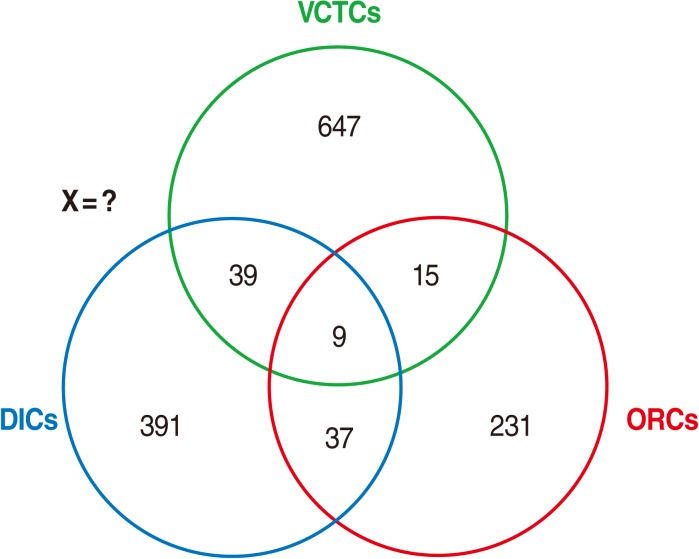
Distribution of intravenous drug users registered by three centers: Volunteer Counseling and Testing Centers (VCTCs); Outreach Teams (ORTs); and Drop in Centers (DICs).

For this purpose, the list of IDUs recorded in these three data sources were extracted and compared with each other, in order to identify common names listed in more than one database. Since the IDUs' national identification codes were not recorded in the data sources, we used several demographic characteristics of the IDUs for comparison, including first name, second name, age, marital status, and region of residence. Then we arranged the data as shown in Figure 1.

The simplest capture-recapture model is the so-called two-sample model [6]. Another capture-recapture method, which was used in this study, is the so-called three-sample model, in which the three incomplete data sources of IDUs were used. In this approach, the capture-recapture method becomes more complicated and includes the following eight possible models as follows (Figure 1):

1. Number of IDUs identified by VCTCs only (A)
2. Number of IDUs identified by DICs only (B)
3. Number of IDUs identified by ORTs only (C)
4. Number of IDUs identified by A and B but not by C (AB)
5. Number of IDUs identified by A and C but not by B (AC)
6. Number of IDUs identified by B and C but not by A (BC)
7. Number of IDUs identified by all three centers (ABC)
8. Number of IDUs studies identified by none of the three centers (X)

There are many elaborate statistical models available for the analysis of three-sample capture-recapture results. The log-linear model, or Poisson regression, is a simple model which easily accommodates the three sources of data and is able to explore dependence between sources and adjust for dependence by including interaction terms in the model [18]. For this purpose, we prepared a dataset with four variables as follows: (a) "var_A", with values 0 or 1, that described belonging to list A; (b) "var_B", with values 0 or 1, that described belonging to list B; (c) "var_C", with values 0 or 1, that described belonging to list C; and (d) "var_freq", which was a non-negative variable that described the frequency of observations in the combination of lists given by variables A, B and C. The unknown frequency of cases occurring in none of the lists was considered as missing. This missing value was estimated by the Poisson model.

The interaction terms were used to model dependence. The basic assumption for the capture-recapture model is that there is no three-list interaction term for three lists. In other words, the third-order interaction vanishes (ABC=0) [18]. By accommodating the three sources of data as shown above, the log-linear model can estimate the number of IDUs not identified by any of the three centers (X) and hence the total population of IDUs (N).

There are two different information criteria which were proposed for model selection: Akaike's Information Criterion (AIC) and the Bayesian Information Criterion (BIC) [19]. The AIC is calculated as follows:

AIC=G^2^-[2×(df)]

Where G^2^ is the likelihood ratio statistic associated with the fit of any model to the data and df denotes the degrees of freedom of the model. The model giving the smallest value of AIC is the one selected. The second criterion, BIC, which is usually preferred to AIC in some applications, is calculated as follows:

BIC=G^2^-[ln (Nobs/2π)]×(df)

Where G^2^ and df are defined as above, and where ln (Nobs) is natural logarithm of the number of parameters in the model. As above, the model giving the smallest value of BIC is the one selected.

All analyses were performed at the 0.05 significance level using statistical software Stata version 11.0 (StataCorp, College Station, TX, USA).

## RESULTS

The general characteristics of the three data sources are shown in Table 1. The majority of IDUs were single men aged 30 to 44 years. Out of 1,478 IDUs identified by three centers, 710 (48%) were identified by VCTCs, 476 (32%) by DICs, and 292 (20%) by ORTs. In addition, 100 (7%) of IDUs were identified by at least two centers and 9 (1%) by all three centers (Figure 1). After exclusion of duplicates, 1,369 IDUs remained.

The results of the log-linear analysis are shown in Table 2. The p-values show that there were significant differences between the saturated model (the 8th model) and all other reduced models. The sixth model (ABC AB BC) was the best fitting model with the smallest value of both AIC and BIC. According to these findings, there might be 9,964 (95% confidence interval [CI], 6,088, 17,636) IDUs who were not identified by any of the centers. Hence, the real number of IDUs is expected to be 11,333 (1,369+9,964). Accordingly, the combined completeness of the three data sources was almost 12% (95% CI, 7% to 18%). Among the three data sources, VCTCs were more complete than the two other centers.

Table 3 shows the completeness of detecting IDUs by the three centers. The completeness of the VCTCs was the highest and that of DICs was the lowest. However, the completeness values for all centers were very low.

## DISCUSSION

Estimation of the size of IDU populations is essential for planning and providing health care services to this group of individuals [12]. In addition, a valid and reliable estimate of the IDU population is needed for estimating the probable outbreaks of blood born infections associated with drug injection such as HIV and HCV [13]. Our findings revealed that the majority of IDUs were not identified by any of the centers.

The p-values showed that there was a significant difference between the reduced models and the saturated one. We used AIC and BIC criteria in order to choose the best fitting model among the models, all of which were statistically significantly different to the saturated model. The sixth model with the smallest value of AIC or BIC was the one selected as the best fitting model. However, care must be taken when using AIC and BIC values for model selection. AIC and BIC do not provide a test of a model in the sense of testing a null hypothesis; i.e. AIC and BIC can tell nothing about how well a model fits the data in an absolute sense. If all candidate models fit the data poorly, these values will not give any warning of this [20].

One might try two-sample methods (AB, AC, and BC) and compare the results with the three-sample method (ABC). However, it is important to remember that the capture-recapture method requires a sufficiently high level of overlapping information to produce a reliable estimate of the unknown population [18]. Using the three-sample approach increases the overlapping area and thus produces more reliable estimates than that of the two-sample approach. Therefore, if possible, the three-sample approach is preferred.

The completeness of the three lists was low and different. The proportion of overlapping cases was relatively low; however, sufficiently high overlapping information is required to produce a reliable estimate of the unknown population [18]. This might result in overestimation of the pooled estimates. This may explain the low completeness of the data sources. Furthermore, the VCTCs, DICs and ORTs provide different services and IDUs may refer to these centers for different purposes. This might explain the difference observed between the completeness of the three centers.

Capture-recapture is a simple statistical approach and represents an attractive method to assess the completeness of different data sources in order to estimate the size of the unknown population, which could not be identified from the data sources. Although the capture-recapture method is a useful and practical approach to estimate the hard-to-reach populations, due to the assumptions and limitations of the method, the results must be interpreted with caution.

A number of studies used the three-sample capture-recapture method to estimate the population of IDUs in other countries. These studies revealed that registries underestimated the real size of the population of IDUs. Davies et al. [21] estimated the number of injecting drug users in the city of Edinburgh, Scotland, in 1999, using the capture-recapture method. The number of IDUs was estimated to be 1,770 from the registry data, while the capture-recapture method estimated the number of IDUs to be 2,070. Accordingly, the completeness of the registry data was 86%. Hickman et al. [22] assessed IDU prevalence in Bristol in 2004. They reported that the total number of IDUs recorded by the three registries was 2,986, whereas the overall estimated number of IDUs using the capture-recapture method was 5,540. Based on these results, the overall completeness of the registries was 54%. Böhning et al. [23] and Goli et al. [16] used a different statistical method, the so-called mixture Poisson model, to estimate the prevalence of heroin consumption in Bangkok and cigarette smokers in Hamadan in the west of Iran, respectively.

Although the capture-recapture approach is a potentially useful method for estimating the size of an unknown population, this method, like any other statistical procedures, has its own limitations. A critical limitation of this method is that sufficiently high overlapping information is required to produce a reliable estimate of the unknown population size. Otherwise, the likelihood functions may become flat and the resulting estimates based on log-linear models may possibly become unstable [18]. Another limitation of the capture-recapture method using log-linear models for investigating an unknown population is that a relative large number of data sources is required to hold the assumption of the normal distribution within log-linear models. Moreover, the validity of capture-recapture results depends on some assumptions. If these assumptions are not considered, the estimates may not be reliable. A critical assumption of capture-recapture methods is the independence of the data sources, so that either positively or negatively dependent sources may cause either underestimation or overestimation of the pooled estimates, respectively [6]. Of course, log-linear models are able to handle dependence among data sources and adjust for this dependence by including interaction terms in the model [18].

Despite its limitations, the current study indicated that the estimated population of IDUs based on registries, and hence the prevalence estimates of IDUs, are considerably underestimated relative to the actual values. Therefore, the policymakers who plan and provide health care services for IDUs should take this issue into special consideration.

The results of this study indicated that the capture-recapture method is an easy to conduct and straightforward approach for the estimation of the size of the hard-to-reach population of IDUs. According to these findings, the majority of the IDUs were not identified by the registry centers. Although the capture-recapture method is a useful and practical approach to estimate unknown populations, due to the assumptions and limitations of the method, the results must be interpreted with caution.

## Figures and Tables

**Table 1:**
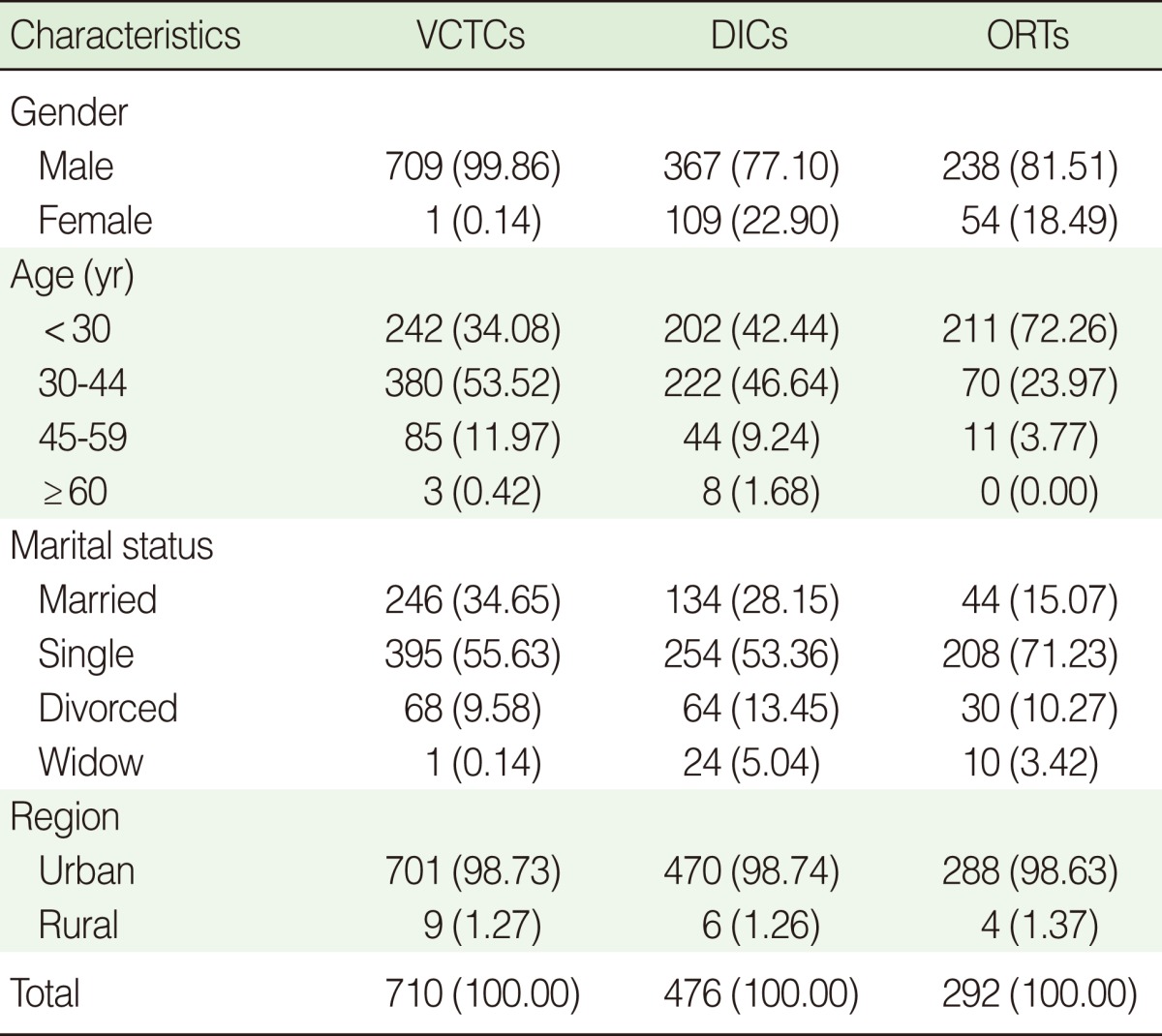
Distribution of the demographic characteristics of the intravenous drug users registered by three centers including Volunteer Counseling and Testing Centers (VCTCs); Outreach Teams (ORTs); and Drop in Centers (DICs)

Values are presented number (%).

**Table 2:**
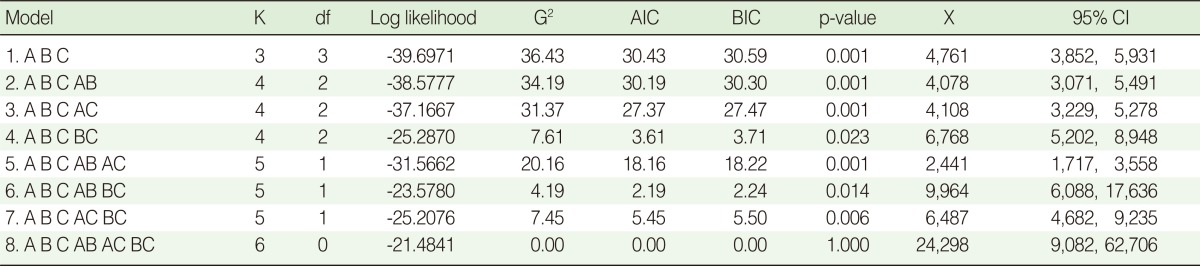
Log-linear models fitted to three lists of intravenous drug users registered by three centers and estimated number of intravenous drug users which was not registered by the centers

K, number of parameters; df, degree of freedom; G2, deviance; AIC, Akaike Information Criterion; BIC, Bayesian Information Criterion; X, unregistered; CI, confidence interval.

**Table 3:**
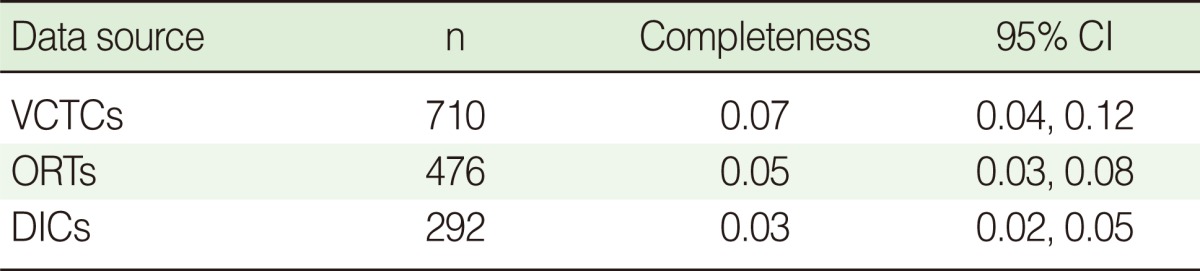
Comparison of the completeness of detecting intravenous drug users by the three centers including Volunteer Counseling and Testing Centers (VCTCs); Outreach Teams (ORTs); and Drop in Centers (DICs)
